# Supplementary material for: Timing of Adiposity Rebound and Determinants of Early Adiposity Rebound in Korean Infants and Children Based on Data from the National Health Insurance Service
Source: Nutrients. 2022 Feb 22;14(5):929. doi: 10.3390/nu14050929 (PMC8912573; doi:10.3390/nu14050929)
Supplement: Supplementary file 1 [file nutrients-14-00929-s001.zip › nutrients-1597517-supplementary.pdf]

Table S1. Body mass index levels (kg/m^2) at each time period (*n*=142,668)

Age at survey (time period)	Boys (*n*=73,389)			Girls (*n*=69,279)		
	Average (all)	High (*n*= 32,572)	Low (*n*=40,817)	Average (all)	High (*n*= 27,404)	Low (*n*=41,875)
4~6 month (1st)	18.08±1.68	18.65±1.68	17.63±1.54	17.57±1.66	18.19±1.67	17.16±1.51
9~12 month (2nd)	17.46±1.47	18.03±1.47	17.00±1.29	16.99±1.46	17.62±1.46	16.57±1.30
18~24 month (3rd)	16.50±1.37	17.10±1.37	16.02±1.16	16.11±1.34	16.79±1.34	15.66±1.13
30~36 month (4th)	16.19±1.29	16.86±1.28	15.65±1.03	15.93±1.32	16.71±1.31	15.42±1.05
42~48 month (5th)	16.09±1.34	16.90±1.29	15.45±0.98	15.92±1.35	16.85±1.29	15.31±0.99
54~60 month (6th)	16.08±1.54	17.14±1.53	15.23±0.89	15.88±1.48	17.05±1.42	15.12±0.92
66~71 month (7th)	16.24±1.88	17.78±1.74	15.01±0.72	15.96±1.73	17.59±1.49	14.90±0.79

Results were presented as means ± standard deviation. H: high BMI group based on the average BMI at the 7th time period; L: low BMI groups based on the average BMI at the 7th time period; M: average BMI group based on the at the 7th time period

Table S2. Detailed distribution (n, %) of body mass index (BMI) percentile for the seven-time periods according to each time period occurring adiposity rebound (AR) among boys

Age at survey (time period)	Percentile	Time period occurring adiposity rebound (AR) among boys (n=73,389)						
		1st (n=1,620)	2nd (n=2,310)	3rd (n=9,478)	4th (n=12,573)	5th (n=12,476)	6th (n=15,123)	non AR (n=19,809)
4~6 month (1st)	5th (n=1,752)	623	80	148	188	189	210	314
		38.46	3.46	1.56	1.50	1.51	1.39	1.59
	5~15th (n=3,532)	365	164	444	525	541	660	833
		22.53	7.10	4.68	4.18	4.34	4.36	4.21
	15~85th (n=45,524)	605	1574	6037	7890	7705	9409	12304
		37.35	68.14	63.69	62.75	61.76	62.22	62.11
	85~95th (n=12,054)	15	297	1575	2091	2140	2558	3378
		0.93	12.86	16.62	16.63	17.15	16.91	17.05
	95th (n=10,527)	12	195	1274	1879	1901	2286	2980
		0.74	8.44	13.44	14.94	15.24	15.12	15.04
9~12 month (2nd)	5th (n=1,993)	47	662	209	231	225	272	347
		2.90	28.66	2.21	1.84	1.80	1.80	1.75
	5~15th (n=4,844)	126	535	712	801	696	854	1120
		7.78	23.16	7.51	6.37	5.58	5.65	5.65
	15~85th (n=50,013)	1144	1060	6546	8614	8426	10510	13713
		70.62	45.89	69.07	68.51	67.54	69.50	69.23
	85~95th (n=10,036)	164	35	1217	1743	1928	2134	2815
		10.12	1.52	12.84	13.86	15.45	14.11	14.21
	95th (n=6,503)	139	18	794	1184	1201	1353	1814
		8.58	0.78	8.38	9.42	9.63	8.95	9.16
18~24 month (3th)	5th (n=1,682)	14	18	1049	132	122	141	206
		0.86	0.78	11.07	1.05	0.98	0.93	1.04
	5~15th (n=3,670)	41	62	1399	482	450	506	730
		2.53	2.68	14.76	3.83	3.61	3.35	3.69
	15~85th (n=48,972)	915	1382	6375	8419	8127	10300	13454
		56.48	59.83	67.26	66.96	65.14	68.11	67.92
	85~95th (n=11,840)	341	452	479	2187	2329	2583	3469
		21.05	19.57	5.05	17.39	18.67	17.08	17.51
	95th (n=7,225)	309	396	176	1353	1448	1593	1950
		19.07	17.14	1.86	10.76	11.61	10.53	9.84
30~36 month (4th)	5th (n=1,443)	10	10	53	892	128	144	206
		0.62	0.43	0.56	7.09	1.03	0.95	1.04
	5~15th (n=3,804)	25	45	263	1627	463	555	826
		1.54	1.95	2.77	12.94	3.71	3.67	4.17
	15~85th (n=51,523)	880	1309	6274	9116	8771	10823	14350
		54.32	56.67	66.20	72.50	70.30	71.57	72.44
	85~95th (n=10,277)	321	443	1636	712	1954	2285	2926
		19.81	19.18	17.26	5.66	15.66	15.11	14.77
	95th (n=6,342)	384	503	1252	226	1160	1316	1501
		23.70	21.77	13.21	1.80	9.30	8.70	7.58
42~48 month (5th)	5th (n=699)	3	5	11	42	444	74	120
		0.19	0.22	0.12	0.33	3.56	0.49	0.61
	5~15th (n=2,543)	10	17	127	210	1184	424	571
		0.62	0.74	1.34	1.67	9.49	2.80	2.88
	15~85th (n=50,547)	789	1102	5493	8016	9558	10926	14663
		48.70	47.71	57.96	63.76	76.61	72.25	74.02
	85~95th (n=11,847)	333	485	1982	2485	982	2499	3081
		20.56	21.00	20.91	19.76	7.87	16.52	15.55
	95th (n=7,753)	485	701	1865	1820	308	1200	1374
		29.94	30.35	19.68	14.48	2.47	7.93	6.94
54~60 month (6th)	5th (n=583)	3	3	10	26	37	381	123
		0.19	0.13	0.11	0.21	0.30	2.52	0.62
	5~15th (n=2,195)	9	6	76	168	230	1098	608
		0.56	0.26	0.80	1.34	1.84	7.26	3.07
	15~85th (n=50,678)	709	1036	5075	7365	8310	12443	15740
		43.77	44.85	53.55	58.58	66.61	82.28	79.46

	85~95th (n=10,906)	325	432	1878	2524	2327	959	2461
		20.06	18.70	19.81	20.07	18.65	6.34	12.42
	95th (n=9,027)	574	833	2439	2490	1572	242	877
		35.43	36.06	25.73	19.80	12.60	1.60	4.43
66~71 month (7th)	5th (n=1,023)	2	3	17	45	63	108	785
		0.12	0.13	0.18	0.36	0.50	0.71	3.96
	5~15th (n=2,853)	17	16	106	195	228	411	1880
		1.05	0.69	1.12	1.55	1.83	2.72	9.49
	15~85th (n=47,834)	662	929	4574	6617	7642	11238	16172
		40.86	40.22	48.26	52.63	61.25	74.31	81.64
	85~95th (n=9,972)	279	386	1690	2267	2285	2216	849
		17.22	16.71	17.83	18.03	18.32	14.65	4.29
	95th (n=11,707)	660	976	3091	3449	2258	1150	123
		40.74	42.25	32.61	27.43	18.10	7.60	0.62

Determination of BMI Percentile by age and gender was based on the child growth standards of the World Health Organization. Precisely, BMI-for-age less than 5th percentile is categorized into severe underweight; 5th to less than 15th percentile into underweight. 15th to less than 85th percentile into healthy weight; 85th to less than 95th percentile into overweight; 95th to 100th percentile into obesity based on the age at the time of measurement.

Table S3. Detailed distribution (n, %) of body mass index (BMI) percentile for the seven-time periods according to each time period occurring adiposity rebound (AR) among girls

Age at survey (time period)	percentile	Time period occurring adiposity rebound (AR) among girls (n=69,279)						
		1st (n=2,041)	2nd (n=3,039)	3rd (n=11,272)	4th (n=12,350)	5th (n=10,332)	6th (n=12,517)	non AR (n=17,728)
4~6 month (1st wave)	5th (n=1,260)	593	64	118	110	101	121	153
		29.05	2.11	1.05	0.89	0.98	0.97	0.86
	5~15th (n=3,296)	448	197	482	457	414	540	758
		21.95	6.48	4.28	3.70	4.01	4.31	4.28
	15~85th (n=46,136)	971	2191	7549	8339	6921	8319	11846
		47.57	72.10	66.97	67.52	66.99	66.46	66.82
	85~95th (n=10,804)	22	374	1784	2013	1682	2053	2876
		1.08	12.31	15.83	16.30	16.28	16.40	16.22
	95th (n=7,783)	7	213	1339	1431	1214	1484	2095
		0.34	7.01	11.88	11.59	11.75	11.86	11.82
9~12 month (2nd wave)	5th (n=1,517)	35	630	194	157	119	158	224
		1.71	20.73	1.72	1.27	1.15	1.26	1.26
	5~15th (n=4,224)	146	715	657	639	532	633	902
		7.15	23.53	5.83	5.17	5.15	5.06	5.09
	15~85th (n=49,666)	1484	1629	8247	9020	7423	9036	12827
		72.71	53.60	73.16	73.04	71.84	72.19	72.35
	85~95th (n=9,327)	250	47	1443	1723	1505	1792	2567
		12.25	1.55	12.80	13.95	14.57	14.32	14.48
	95th (n=4,545)	126	18	731	811	753	898	1208
		6.17	0.59	6.49	6.57	7.29	7.17	6.81
18~24 month (3th wave)	5th (n=1,180)	7	13	804	91	57	76	132
		0.34	0.43	7.13	0.74	0.55	0.61	0.74
	5~15th (n=3,143)	31	56	1440	360	309	338	609
		1.52	1.84	12.78	2.91	2.99	2.70	3.44
	15~85th (n=49,521)	1240	2018	8311	8983	7193	8962	12814
		60.75	66.40	73.73	72.74	69.62	71.60	72.28
	85~95th (n=9,977)	397	577	558	1893	1802	2028	2722
		19.45	18.99	4.95	15.33	17.44	16.20	15.35
	95th (n=5,458)	366	375	159	1023	971	1113	1451
		17.93	12.34	1.41	8.28	9.40	8.89	8.18
30~36 month (4th)	5th (n=1,307)	8	13	78	830	98	110	170
		0.39	0.43	0.69	6.72	0.95	0.88	0.96
	5~15th (n=3,734)	34	62	314	1672	414	481	757
		1.67	2.04	2.79	13.54	4.01	3.84	4.27
	15~85th (n=50,137)	1185	1910	7807	9081	7563	9284	13307
		58.06	62.85	69.26	73.53	73.20	74.17	75.06
	85~95th (n=9,243)	404	570	1875	600	1564	1797	2433
		19.79	18.76	16.63	4.86	15.14	14.36	13.72
	95th (n=4,858)	410	484	1198	167	693	845	1061
		20.09	15.93	10.63	1.35	6.71	6.75	5.98
42~48 month (5th)	5th (n=629)	6	6	26	35	390	63	103
		0.29	0.20	0.23	0.28	3.77	0.50	0.58
	5~15th (n=2,500)	13	24	152	269	1071	368	603
		0.64	0.79	1.35	2.18	10.37	2.94	3.40
	15~85th (n=50,647)	1111	1717	7267	8776	8205	9630	13941
		54.43	56.50	64.47	71.06	79.41	76.94	78.64
	85~95th (n=10,460)	456	698	2385	2198	564	1846	2313
		22.34	22.97	21.16	17.80	5.46	14.75	13.05
	95th (n=5,043)	455	594	1442	1072	102	610	768
		22.29	19.55	12.79	8.68	0.99	4.87	4.33
54~60 month (6th)	5th (n=552)	3	2	19	30	37	341	120
		0.15	0.07	0.17	0.24	0.36	2.72	0.68
	5~15th (n=2,734)	20	27	130	228	268	1289	772
		0.98	0.89	1.15	1.85	2.59	10.30	4.35
	15~85th (n=52,225)	1114	1774	7341	8723	7954	10359	14960
		54.58	58.37	65.13	70.63	76.98	82.76	84.39
	85~95th (n=8,800)	407	608	2134	2160	1501	464	1526
		19.94	20.01	18.93	17.49	14.53	3.71	8.61
	95th (n=4,968)	497	628	1648	1209	572	64	350
		24.35	20.66	14.62	9.79	5.54	0.51	1.97
66~71 month (7th)	5th (n=840)	3	4	29	42	40	68	654
		0.15	0.13	0.26	0.34	0.39	0.54	3.69

5~15th (n=3,293)	20	25	144	232	251	479	2142
	0.98	0.82	1.28	1.88	2.43	3.83	12.08
15~85th (n=50,300)	1042	1629	6959	8256	7656	10234	14524
	51.05	53.60	61.74	66.85	74.10	81.76	81.93
85~95th (n=8,615)	422	616	2107	2177	1579	1344	370
	20.68	20.27	18.69	17.63	15.28	10.74	2.09
95th (n=6,231)	554	765	2033	1643	806	392	38
	27.14	25.17	18.04	13.30	7.80	3.13	0.21

Determination of BMI Percentile by age and gender was based on the child growth standards of the World Health Organization. Precisely, BMI-for-age less than 5th percentile is categorized into severe underweight; 5th to less than 15th percentile into underweight. 15th to less than 85th percentile into healthy weight; 85th to less than 95th percentile into overweight; 95th to 100th percentile into obesity based on the age at the time of measurement.
